# Draft Genome Sequences of Four Enterococcus cecorum Strains Isolated from American Black Vultures (Coragyps atratus) and Harris’s Hawk (Parabuteo unicinctus) in Rio de Janeiro, Brazil

**DOI:** 10.1128/mra.01361-22

**Published:** 2023-04-12

**Authors:** Andréa Andrade Rangel Freitas, Stephanie Silva Rodrigues Souza, Adriana Rocha Faria, Paul J. Planet, Vânia Lúcia Carreira Merquior, Lúcia Martins Teixeira

**Affiliations:** a Instituto de Microbiologia Paulo de Góes, Universidade Federal do Rio de Janeiro, Rio de Janeiro, RJ, Brazil; b Faculdade de Ciências Médicas, Universidade do Estado do Rio de Janeiro, Rio de Janeiro, RJ, Brazil; c Faculdade de Medicina, Universidade Federal do Rio de Janeiro, Rio de Janeiro, RJ, Brazil; d Perelman School of Medicine, University of Pennsylvania, Philadelphia, Pennsylvania, USA; e Pediatric Infectious Disease Division, Children’s Hospital of Philadelphia, Pennsylvania, USA; f Sackler Institute for Comparative Genomics, American Museum of Natural History, New York, New York, USA; University of Arizona

## Abstract

We report the draft genome sequences of four Enterococcus cecorum strains obtained from cloacal swab specimens collected from three healthy captive wild birds (two Coragyps atratus and one Parabuteo unicinctus) in Rio de Janeiro, Brazil. The genome sizes ranged from 2.38 to 2.55 Mb.

## ANNOUNCEMENT

Enterococcus cecorum is a commensal of the intestinal microbiome of mammals and birds (1), particularly among healthy adult chickens (2–4). Nonetheless, it has been increasingly recognized as an emerging pathogen for broiler and broiler breeder chickens in Europe and North America (2–7).

Here, we report the draft genome sequences of four *E. cecorum* strains isolated from two American black vultures (Coragyps atratus, named ABV-1 and ABV-2) and one Harris’s Hawk (Parabuteo unicinctus, designated HH) (Table 1). The birds were admitted to a wildlife rehabilitation center in Rio de Janeiro, Brazil, and had their microbiota examined during an investigation on the carriage of antimicrobial-resistant bacteria by wild birds. Cloacal content samples were collected using swabs (Transystem with Amies medium; Copan, Brescia, Italy) and homogenized in 1 mL of STGG broth (8) each. Then, 100-μL aliquots were plated into different culture media (Table 1), followed by incubation at 37°C for 18 to 24 h. Bacterial strains were identified by matrix-assisted laser desorption ionization–time of flight mass spectrometry (MALDI-TOF MS), using Microflex equipment, according to the manufacturer’s recommendations (Bruker Daltonics, Bremen, Germany), by direct transfer of an isolated colony to a spot of the target plate (9).

**TABLE 1:** Summary of information on the origin, isolation, and genomic metrics of four Enterococcus cecorum strains isolated from wild birds in Rio de Janeiro, Brazil

Parameter	Enterococcus cecorum strain			
	ESM13C1	ESM13AIC1	E877AIC1	E1062
Host bird/source of isolation	HH^*a*^	HH^*a*^	ABV-1^*b*^	ABV-2^*b*^
Culture medium used for isolation	Enterococcosel agar^*c*^	Columbia agar^*c*^ with 10% inulin^*d*^	Columbia agar^*c*^ with 10% inulin^*d*^	CHROMagar^*c*^
Genome size (bp)	2,557,983	2,553,798	2,382,250	2,524,751
Raw reads	1,470,976	1,465,612	1,768,236	1,505,218
G+C content (%)	35.98	35.98	36.25	36.03
No. of contigs	104	97	83	151
Mean coverage (×)	122	116	129	123
No. of CDSs^*e*^	2,565	2,555	2,403	2,601
*N*_50_ (bp)	58,299	63,768	97,499	37,638
No. of tRNAs	55	55	57	52
No. of rRNAs	4	3	2	3
SRA accession no.	SRR18070396	SRR18070395	SRR18070394	SRR18070397
NCBI assembly no.	JAKUDT000000000	JAKUDS000000000	JAKUDR000000000	JAKUDU000000000

a Harris’s Hawk (Parabuteo unicinctus).

b American black vulture (Coragyps atratus).

c BD Microbiology Systems, USA.

d Sigma Co, USA.

e CDSs, coding sequences.

For DNA extraction, strains were grown on blood agar plates (Plastlabor, Rio de Janeiro, Brazil) for 18 to 24 h at 37°C with 5% CO_2_, and a single bacterial colony was inoculated into 5 mL of tryptic soy broth. After overnight incubation at 37°C, an aliquot of 1.5 mL of each culture was used for preparation of genomic DNA for sequencing by using a Wizard genomic DNA purification kit (Promega, Madison, WI, USA). Paired-end (125-bp read) libraries were prepared using the Nextera XT DNA sample preparation kit (Illumina, Inc., CA) and sequenced on a HiSeq 2500 sequencer (Illumina Inc., San Diego, CA, USA) to yield a total of reads and base pairs specified in Table 1. FastQC 0.11.8 (10) was applied to analyze read quality. Reads were trimmed (Trim Galore v.0.6.2) (11), assembled (Unicycler v0.4.8) (12), and annotated (RAST tool kit [RASTtk]) (13) using the resources of the Pathosystems Resource Integration Center (PATRIC 3.6.10) (14). Genomic metrics data for the four sequences are summarized in Table 1. Default parameters were used for all software tools.

Antimicrobial resistance genes were identified by using the ResFinder 4.1 tool (15). All strains harbored *tet*(M) (tetracycline resistance), while strains ESM13C1 and ESM13AIC1 also presented *tet*(L). In comparison to the other strains, ESM13C1 and ESM13AIC1 contained *erm*(B) (macrolide, lincosamide, and streptogramin B resistance) and *vatE* (streptogramin A resistance) genes. Additional lincosamide resistance genes were also observed: *Inu*(G) in E1062 and *Inu*(C) in E877C1, ESM13C1, and ESM13AIC1. No virulence-associated genes were identified by using VirulenceFinder 2.0 (16). PlasmidFinder 2.1 (17) identified repUS43 in both E1062 and E877AI strains. PHASTER (18) predicted one intact prophage region (PHAGE_Strept_A25_NC_028697) in E1062. CRISPRCasFinder (19) identified sequences associated with CRISPR in E877AIC1 (*n* = 2) and in ESM13AIC1 (*n* = 1).

### Data availability.

The four whole-genome shotgun projects have been deposited at DDBJ/ENA/GenBank under the SRA accession numbers SRR18070394, SRR18070395, SRR18070396, and SRR18070397 and NCBI assembly accession numbers JAKUDR000000000, JAKUDS000000000, JAKUDT000000000, and JAKUDU000000000 for strains E877AIC1, ESM13AIC1, ESM13C1, and E1062, respectively. Raw sequence reads (Table 1) have been deposited in the NCBI Sequence Read Archive (SRA) under the BioProject accession number PRJNA808242.
